# Development and preliminary psychometric properties of the General Practitioner Attitudes and Confidence Scale (GPACS–D) for dementia

**DOI:** 10.1186/s12875-016-0506-z

**Published:** 2016-08-04

**Authors:** Ron L. Mason, Michael J. Annear, Amanda Lo, Fran McInerney, Laura T. Tierney, Andrew L. Robinson

**Affiliations:** 1 Wicking Dementia Research and Education Centre, University of Tasmania, Private Bag 143, Hobart, TAS 7001 Australia; 2 School of Medicine, Faculty of Health, University of Tasmania, Private Bag 34, Hobart, TAS 7001 Australia; 3 School of Health Sciences, Faculty of Health, University of Tasmania, Private Bag 135, Hobart, TAS 7001 Australia

**Keywords:** Attitudes, Confidence, Dementia education, General practitioner, Scale psychometrics

## Abstract

**Background:**

International evidence suggests that dementia is under–diagnosed in the community and that General Practitioners (GPs) are often reluctant to engage to their fullest capability with patients who exhibit cognitive symptoms. This is potentially reflected by a lack of GP knowledge about the syndrome. However, it is also recognised that attitudes and confidence are important in relation to how and to what extent a GP approaches a person with dementia. This research sought to develop a reliable and valid measure of GPs attitudes and confidence towards dementia.

**Methods:**

The General Practitioner Attitudes and Confidence Scale for Dementia (GPACS–D) was developed via a four stage process, including initial content development, pretesting, pilot testing and psychometric evaluation, including Principal Component Analysis (PCA). Participants were recruited for pre–testing (*n* = 12), test–retest (*n* = 55), and dementia workshop pre–and post–education evaluation (*n* = 215).

**Results:**

The process of scale development and psychometric evaluation resulted in a 20-item measure of GP attitudes and confidence towards dementia, with 4 items removed due to poor reliability, low sensitivity, or lack of model fit. Among 55 respondents who completed the scale on two occasions with no intervening education, Kappa coefficient scores per item ranged from fair (*n* = 2, candidates for removal), moderate (*n* = 5), substantial (*n* = 15), and almost perfect (*n* = 2). A test of the sensitivity of item scores to change following dementia education among 215 GPs indicated that, with the exception of one item, all scale responses exhibited significant differences between pre–and post–workshop scores, indicating acceptable sensitivity. With one further item removed due to a low communality score, the final PCA undertaken with the remaining 20 items supports a four–component solution, which accounted for 51.9 % of the total variance.

**Conclusion:**

The GPACS–D provides a reliable and preliminarily valid measure of GP attitudes and confidence towards dementia. The scales provide useful information for medical educators and researchers who are interested in evaluating and intervening in GP perceptions of the syndrome and their capacity to provide effective care.

## Background

Dementia is underdiagnosed in Australia and comparably developed countries. Many older people with this syndrome have not received a formal diagnosis from a health practitioner [1]. It is widely acknowledged that general practitioners (GPs) are pivotal in facilitating early diagnosis of dementia and that their attitudes and confidence towards identification, treatment, and management of the syndrome play a key role in influencing dementia diagnosis and care [2]. Reported reluctance among some GPs to identify dementia and to make a formal diagnosis, or refer to an appropriately qualified medical specialist [3], may reflect their negative attitudes towards the syndrome [4], as well as a lack of confidence in their ability to diagnose suspected dementia [5, 6].

Attitudes towards dementia have been reported as key determinants of physician engagement with a possible presentation of dementia [7]. For example, Cahill et al. [8, p665] report that “therapeutic nihilism” (the belief that there is no treatment or cure for dementia) means that GP’s who hold this perception see no value or advantage to early diagnosis. Research underpinned by social psychological theories has focused particularly on how attitudes are formed and maintained and how they impact on behavioural intention, decisions to act, or actual clinical or health behaviours [8]. Whether an attitude is favourable or unfavourable has been reported to influence the type of response to the object of that attitude [8]–such as dementia. Boise et al. [7] found that among physicians, attitude towards dementia is a key determinant of whether or not they conduct a detailed assessment of the patient.

Similarly, social psychological theories [9, 10] suggest that an association exists between confidence and behaviour within health care and other settings [11]. An individual’s behaviour is influenced by confidence in their ability to perform a particular set of actions or procedures. Bandura [10] refers to this as *self*-*efficacy*, while Ajzen [12] describes it as *perceived behavioural control*. Both terms are concerned with an individuals’ perceived ability to perform a particular behaviour and the impact of this perception on their intentions to act or actual practice [9]. Research suggests that levels of confidence in one’s ability to diagnose, treat, or manage dementia may be correlated with the quality of care received [13]. The above theoretical framework is relevant to this study as it is estimated that one third of GPs lack confidence in their diagnostic skills, while two thirds lack confidence in the management of behaviours associated with dementia [14].

A GP’s response to a patient presenting with possible dementia is, therefore, not only influenced by their attitudes towards the syndrome but also by perceptions of their ability to perform a diagnosis and assist in the management of the syndrome. We contend that attitudes and confidence may well be positioned within a singular measure as a theoretical construct (a precursor to behaviour), which may give rise to relevant subscales within this domain. Research indicates that there is a positive correlation between self–estimated confidence and general attitudes towards caring for people with dementia. For example, Kaduszkiewicz et al. [15] found that physicians with a negative attitude towards caring for patients with dementia reported a lack of belief in their ability to improve the patients’ quality of life, while the opposite was true for those with a positive attitude [16]. Such findings provide support for the conceptualisation of attitudes and confidence as co–related influences on how a GP may be expected to engage with a person with dementia.

Few studies have explored the co–related concepts of attitudes and confidence using a valid and reliable instrument. Limited research has reported on GPs general attitudes and practices towards screening and disclosing a diagnosis to patients with dementia [14, 16], comparative analyses of GP attitudes to early diagnosis [2], and self–reported competence and attitudes of GP’s towards patients with dementia [15]. While a small number of survey tools exist to measure GP attitudes and to a lesser extent confidence, no instrument has been used across multiple studies and few have been administered with a pre–test post–test research design, in a dementia education context for example. There is also a notable absence of reports concerning the psychometric performance of such measures.

In relation to dementia education programs, which attempt to improve attitudes and confidence by increasing clinician knowledge, no attempt to measure changes prior to and after an intervention have been undertaken and reports of the psychometric properties of measures are limited. As reported by Liu et al. [17, p14], “there are no studies on how dementia training affects the confidence and attitudes of physicians”. The aim of the current research, therefore, was to develop a reliable, valid, and responsive scale that measures GP attitudes and confidence in relation to the diagnosis, treatment, and management of dementia at baseline and after a targeted educational intervention.

## Methods

The GP Attitudes and Confidence Scale–Dementia (GPACS–D) was developed via a four stage process, including initial content development, pretesting, pilot testing and psychometric evaluation. This process is consistent with published scale development procedures [17]. Throughout the development of the scale, refinement and item reduction was informed by expert feedback and an analysis of pilot results.

### Sampling and scale development

#### Stage 1: Sampling and recruitment

Two cohorts of purposively selected expert participants were recruited to inform item content and construction. Twelve GPs comprised a *focus group*, while eight health professionals and academics comprised the project team and included GPs, medical educators, nurse academics, and social scientists. The focus group and research team participants were recruited via email invitation through local training and organisational networks. A convenience sample of 55 respondents comprising 38 final year medical students and 17 GPs, was recruited by letter of invitation from a School of Medicine at an Australian University to take part in a test–retest procedure to confirm the reliability of survey items. Surveys were administered in lecture time for students and via email for GP’s, who completed and returned the (Time 1) T1 survey. Time 2 (T2) surveys were sent two weeks after participants had completed the T1 survey. In the subsequent pilot study, 215 GPs participated in a dementia–related workshop, conducted throughout Australia between January and September 2015, and completed the GPACS–D before and after the education. The three–hour workshop involved lectures, video case studies, group discussions, and role playing. All workshop participants were invited to complete the GPACS–D before and after the education intervention. Participants were provided with information about the research, and completion of the survey implied consent. A University Human Research Ethics Committee reviewed and approved this study (Reference Number: H0012046). Completed pre–test surveys were collected prior to the workshop, while post–test surveys were collected directly after.

#### Stage 2: Content development

Scale content was developed from information obtained during a scoping literature review from which a pool of 24 potential scale items was compiled [2, 6, 15, 16, 18–20]. Items were sent to the project team for critical review and refinement (RM, AL, AR, MA, FM), focusing on question construction, interpretability, and relevance. Editing and refinement of survey items was iterative and achieved by sending successive versions of the draft instrument to each member of the project team for feedback. The preliminary version of the GPACS_D comprised 24 declarative statements and employed a five–point, Likert–type scale with responses varying from *strongly disagree* to *strongly agree*, which measured respondent sentiment in relation to the topic areas of, early diagnosis, the efficacy of treatment, resources and support, as well as perceived confidence in relation to the diagnosis, treatment and management of dementia.

#### Stage 3. Pretesting

Pretesting was conducted to establish the face and content validity of the tool and included the use of the focus group (referred to above) of 12 GPs, who completed the draft survey and commented on item construction, content appropriateness, relevance and breadth of scope. Feedback provided by the focus group was collated under each survey item and presented to the expert panel for critical review, from which the 24–item scale was developed (Table 1).

**Table 1:** Scale Item Content

Item Number	Item Content
1	Much can be done to improve the quality of life for people with dementia
2	I prefer to have nothing to do with the care of dementia patients
3	Managing dementia is more often frustrating than rewarding
4	The early detection of dementia benefits the patient
5	It is important for relative/family carers of people with dementia to seek external support
6	Relatives/family carers of patients with dementia should be encouraged to contact Alzheimer’s Australia
7	GP’s are in the best position to help relatives/carers with organising care for someone with dementia
8	I fear communicating a diagnosis of dementia will damage the doctor patient relationship
9	Guidelines for the management of dementia would greatly assist in providing care
10	I prefer treating patients with other chronic diseases such as diabetes mellitus or hypertension
11	The term ‘dementia’ should be avoided when discussing a diagnosis with a carer/family member as it is likely to cause emotional distress
12	Patients with dementia should be informed early so they can plan for the future
13	It is important to inform the person with dementia of the terminal course of the condition
14	It is important to inform the relatives/family carers of the person with dementia of the terminal course of the condition
15	I feel frustrated because I do not know how to effectively treat people with dementia
16	Dementia is better treated by specialist physicians
17	I feel confident in my ability to discuss legal issues associated with a diagnosis of dementia
18	I feel confident in my ability to diagnose dementia
19	I feel confident in my ability to communicate a diagnosis of dementia to a patient
20	I feel confident in my ability to provide appropriate medical care for a person with dementia
21	I feel confident in my ability to provide advice about managing dementia related symptoms
22	I feel confident in my ability to provide advice about managing risky behaviours associated with dementia
23	I feel confident in my knowledge of local resources to assist families/carers caring for a person with dementia
24	A geriatrician review is essential in any definitive diagnosis of dementia

#### Stage 4: Pilot administration

In order to evaluate the test–retest reliability of the GPACS–D, the tool was administered to 55 respondents (38 final year medical students and 17 GPs), with two weeks elapsing between the first and second administration, and no intervening education. A diversity of student and practicing medical practitioners was desirable during test–retest administration as the GPACS–D is intended for use at all stages of a medical career. The survey was also administered at a series of dementia education workshops to determine the internal consistency and construct validity of the measure. In total, 215 GPs completed the survey prior to and directly after the workshop.

#### Stage 5: Evaluation of psychometric properties and instrument refinement

A process of item evaluation and reduction was undertaken, through an examination of pilot responses to ensure that only the most reliable and valid items were included in the scale. Following the initial removal of items that exhibited poor reliability and sensitivity, Principal Component Analysis (PCA) was performed to further refine the scale, determine preliminary factorial validity, and identify potential subscales within the measure.

### Analysis

All data analyses were conducted using SPSS (Version 20) [21]. Considering the level of measurement (ordinal), non–parametric measures of intra–rater reliability were an appropriate analytic approach. A weighted Kappa (k) coefficient was used to provide a measure of test–retest reliability at item level. Weighted Kappa is appropriate for use with ordinal data and is equivalent to the intra–class correlation coefficient [22]. The following have been identified as standards for strength of agreement for Kappa coefficients: 0 = ‘poor’; .01–.20 = ‘slight’; .21–.40 = ‘fair’; .41 to .60 = ‘moderate’; .61–.80 = ‘substantial’; .81–1.00 = ‘almost perfect’ [23, 24]. A Kappa coefficient above .40 was selected as the cut–off for item selection as suggested by Fleiss et al. [25]. Expected and observed agreement levels are also reported. Items with a Kappa coefficient above .40 and an observed agreement score of 90 % or better were deemed reliable [26] and retained, while items scoring below any of these criteria were excluded. A Wilcoxon signed ranks test for paired samples was undertaken to identify potentially significant differences between survey item scores obtained from the pilot study to assess each item’s sensitivity to change (a measure of construct validity) following educational intervention (dementia workshop). Items exhibiting a non–significant difference between pre and post–education administration were identified as candidates for elimination. One item, *I prefer to have nothing to do with the care of dementia patients* was eliminated as a result. An exploratory analysis employing PCA was undertaken (with Varimax rotation) to identify any patterns among variables. Any items exhibiting poor fit were eliminated at this stage of the analysis with the PCA being re–run to produce a final factor solution. Finally, tests of internal consistency utilising a Cronbach’s Alpha (α) value was calculated for each of the potential sub scales indicated by the PCA.

## Results

In total, 270 GPs participated in pilot testing the GPACS–D measure, including 55 respondents in a test–retest administration and 215 who completed the scale before and after a dementia workshop. The workshop sample comprised 3 % medical educators (*n* = 8), 65 % GP Registrars (*n* = 139), 32 % GPs (*n* =68). The mean age of respondents was 39.3 (*SD* = 11.4), 63 % were born in Australia (*n* = 132), and 66 % were female (*n* = 92). A 100 % response rate for the test retest phase and a 93 % response rate for the pilot testing workshop were achieved. The very high level of participation among the GP cohort undertaking the workshop suggests that the samples are likely to be representative of Australian GPs who are undergoing specialised GP post–graduate training.

### Test retest reliability

Among 55 respondents to the GPACS–D who completed the scale on two occasions with no intervening education, Kappa coefficient scores ranged from ‘fair’ to ‘almost perfect’ reliability [23], with 0 items rated as ‘poor’, 2 items rated ‘fair’, 5 items rated ‘moderate’, 15 items rated ‘substantial’, and 2 items rated ‘almost perfect’. Additionally, observed agreement between time one and time two scores was significantly higher than expected agreement, with over 90 % agreement scored on all items [26]. These results indicate good test–retest reliability with the exception of two items: Item 19: *I feel confident in my ability to communicate a diagnosis of dementia to a patient*, and Item 22: *I feel confident in my ability to provide advice about managing risky behaviours* (e.g. *driving*, *wandering*), where coefficient scores were below the inclusion criteria of .40 cited above. These two items were eliminated due to poor reliability. See Table 2: Results of test–retest and pre and post workshop pilot testing.

**Table 2:** Results of test-retest and pre and post workshop pilot testing

	Test-Retest Results						Pilot results for individual survey items – pre and post workshop			
Survey Item	Kappa^a^	SE Kappa	CI 95 %	Expected Agreement (%)	Observed Agreement (%)	‘p’^b^	Pre-Workshop Mean (*n* = 215)	Post-Workshop Mean (*n* = 215)	Wilcoxon Paired Samples Test	
									‘z’	‘p’
1	.707	.140	.430–.980	84.8	95.6	.0000	4.1	4.6	5.769	.000
2	.781	.138	.511–1.00	74.9	94.5	.0000	1.7	1.6	.754	.451
3	.577	.138	.270–.847	90.6	96.0	.0000	2.8	2.4	6.264	.000
4	.722	.140	.450–.994	88.8	96.9	.0000	4.2	4.6	6.313	.000
5	.558	.141	.282–.833	91.0	96.0	.0000	4.5	4.7	4.582	.000
6	.544	.127	.306–.792	90.3	95.6	.0000	4.4	4.6	5.256	.000
7	.639	.140	.365–.913	86.5	95.1	.0002	4.0	4.4	6.391	.000
8	.511	.138	.241–.781	89.6	94.9	.0001	2.0	1.6	4.331	.000
9	.686	.134	.424–.948	92.8	97.8	.0000	4.2	4.4	3.752	.002
10	.764	.141	.487–1.00	88.4	97.3	.0000	3.1	2.6	6.016	.000
11	.704	.137	.436–.972	92.4	97.8	.0000	1.9	1.5	5.673	.000
12	.685	.138	.415–.995	94.4	98.3	.0000	4.3	4.7	6.655	.000
13	.877	.140	.603–1.00	87.9	98.5	.0000	3.6	4.1	8.365	.000
14	.884	.139	.612–.995	90.3	98.9	.0000	4.0	4.7	9.055	.000
15	.723	.139	.451–.995	80.0	94.4	.0000	3.4	2.3	9.860	.000
16	.669	.141	.364–.945	89.1	96.4	.0000	2.8	2.1	7.352	.000
17	.640	.139	.368–.912	87.8	95.6	.0000	2.5	3.4	8.671	.000
18	.628	.139	.356–.900	90.6	96.5	.0000	2.9	3.9	10.770	.000
19	.374	.140	.098–.650	93.4	95.9	.0040	3.2	4.1	9.883	.000
20	.632	.141	.403–.908	91.2	96.8	.0000	3.1	4.0	10.061	.000
21	.679	.141	.403–.995	83.4	94.7	.0000	2.9	3.9	10.398	.000
22	.388	.138	.116–.660	90.2	94.0	.0026	2.9	3.7	9.599	.000
23	.517	.140	.243–.791	83.4	92.0	.0001	2.6	3.6	9.828	.000
24	.694	.141	.418–.970	88.2	96.4	.0000	3.4	2.3	10.306	.000

^a^Weighted Kappa (quadratic)

^b^Significant at .001 level

### Construct validity: sensitivity to change

Two hundred and fifteen participants completed the GPACS–D before and after dementia education. With the exception of one item, Item 2: *I prefer to have nothing to do with the care of dementia patients*, all responses to scale statements exhibited significant differences between pre–and post–workshop scores, indicating both acceptable and hypothesized sensitivity to change. The item that showed no significant change following dementia education was eliminated. (See Table 2).

### Principal component analysis

We undertook a Principle Component Analysis (PCA) using baseline data obtained from 215 participants prior to the educational intervention. A total of 21 items were retained for the PCA. Preliminary analysis confirmed the factorability of the data set (KMO = .811, Bartlett’s test of Sphericity *p* < .001). One item, Item 24: *a geriatrician review is essential in any definitive diagnosis of dementia*, exhibited a low communality score and was removed from the analysis, indicating that the item did not fit well in the solution. The final PCA, undertaken with the remaining 20 items, identified the presence of four components with eigenvalues exceeding 1.0, explaining 23.1 %, 14.6 %, 7.8 % and 7.5 % of the variance respectively, with the four–factor solution accounting for 51.9 % of the total variance. Varimax rotation was used because during the survey design stage, we assumed that the constructs ‘confidence’ and ‘attitudes’ would likely be statistically discrete (independent of each other), and although they might be theoretically related (as highlighted by theories of behaviour change for example), our interest lay in identifying whether those items loading on ‘attitudes’ were distinct from those loading on ‘confidence’. Varimax rotation indicated that 5 to 8 variables loaded significantly on each component. Loadings of < .30 was employed as the cut-off.

Results indicate the presence of four potential sub scales within the measure, which have acceptable factorial validity. Low to moderate correlation between components (.01 to .28) supports the potential presence of distinct subscales. The four identified components were examined by the project team and conceived as: a) *confidence in clinical abilities*; b) *support for quality of life and care*; c) *fears and frustrations*; and d) *communication about dementia progression*. The loadings and interpretation of components indicate that the survey has acceptable preliminary factorial validity.

### Internal consistency

The internal consistency of the 4 subscales identified in the GPACS-D via PCA was obtained using Cronbach’s alpha. Internal consistency of each of the hypothesised subscales ranged from .62 to .89, which is generally indicative of moderate to good internal consistency [27]. See Table 3: Final PCA results.

**Table 3:** Final PCA results

Survey Item	Confidence in clinical abilities	Support for quality of life and care	Communication about dementia progression	Fears and frustrations
1.		.**622**		
3.		.327		.**466**
4.		.**560**		
5.		.**522**		
6.		.**704**		
7.		.**570**		
8.				.**782**
9.	.304	.**451**		
10.	.310			.**426**
11.				.**745**
12.		.457	.**516**	
13.			.**849**	
14.			.**867**	
15.	.**562**		.384	.381
16.	.**612**			
17.	.**755**			
18.	.**776**			
20.	.**807**			
21.	.**826**			
23.	.**713**			
Eigen Values for Component	4.62	2.92	1.50	1.56
Variance explained	23.1	14.6	7.47	7.76
Cronbach’s Alpha	.886	.616	.741	.633

Extraction Method: Principal Component Analysis

Rotation Method: Varimax with Kaiser Normalisation

Bold text indicates those items defining each of the identified factors

## Discussion

Findings from this study demonstrate sound psychometric properties of the 20-item GPACS-D. During the analysis, four items were removed from the original 24-item preliminary version based on the exclusion criteria (poor test-retest reliability, non-significant sensitivity to change, and low communality score in the PCA). Test-retest reliability was confirmed among individuals who had not undertaken specific dementia training or education. Face and content validity was achieved through a scoping review of contemporary literature, focus group discussions with GPs, and pretesting with an expert panel. Construct validity (sensitivity to change) was confirmed through analysis of the results of GPACS-D administration before and after a dementia education workshop. Preliminary factorial validity was supported by the interpretability of PCA results, which indicated that the GPACS-D fits an interpretable four-factor solution. Specific components of the scale include a) confidence in clinical abilities, b) support for quality of life and care, c) communication about dementia progression, and d) fears and frustrations. GPACS-D factor scores display moderate to good internal consistency suggesting they reflect underlying constructs.

The GPACS-D is among the first scales to provide a theoretically informed measure of dementia-related attitudes and confidence that is suitable for administration with medically trained individuals in Australia. Previous studies have reported that attitudes towards particular health conditions and confidence in one’s ability to diagnose, treat, and manage these are correlative with the resultant behaviours and practices of medical professionals [28]. Moreover, when negative attitudes and a lack of confidence has been reported in the literature, GP behaviours towards particular health conditions have been identified as less than optimal [16]. These results are consistent with social psychological theories associated with health and treatment behaviour [9]. These theoretical perspectives suggest that attitude affects the way in which an individual approaches the object of that attitude (in this instance, a patient with suspected dementia). Moreover, confidence (often defined as self-efficacy or perceived behavioural control) is considered vital in relation to the extent to which a GP engages a patient who may be presenting with symptoms of dementia. The GPACS-D provides a new mechanism to measure the attitudes and confidence levels of GPs at baseline as well as following dementia-specific educational interventions.

Counteracting ingrained attitudes associated with diagnosing, and treating people with dementia [4] is a significant task for medical educators. The key to countering such attitudes and, therefore, improving recognition of dementia, is arguably targeted educational programs [3]. Targeted educational interventions aim to increase awareness and inform participants about a particular subject matter, in this case the diagnosis and management of dementia. Scales that accurately measure change in attitudes and confidence are fundamental because the manner in which dementia is approached and managed relies on far more than an individual’s knowledge about the subject (although knowledge is conceptually related to both attitude and confidence) [7]. Additionally, a scale that accurately measures changes in attitudes and confidence provides a basis from which to conduct research that aims to elucidate whether improving attitudes and confidence can affect positive change in the treating behaviour of medical practitioners.

### Limitations

The GPACS-D has been developed with an Australian cohort of GPs and more work is required with an international cohort of medical professionals to validate the scale for a global population. There is also likely to be some variation in attitudes towards and confidence associated with dementia between medical professionals in more and less developed countries where levels of exposure to the syndrome vary based on prevalence. It may also be possible to develop a version of the GPACS-D that is valid and reliable when administered with a wider population of health professionals who routinely interact with people who have dementia (such as nurses and allied health professionals).

A confirmatory factor analysis (CFA) is required to validate the four hypothesised subscales that were identified via the PCA. While the four subscales appear consistent with the theory and literature, they cannot yet be considered valid sub-measures. It is hypothesised that scoring of the GPACS-D will be most effective when summaries can be derived for the four subscales as these potentially measure distinct (though co-related) constructs related to attitudes and confidence. The approach to scoring the GPACS-D that is recommended with the present version is an item level analysis for use with baseline data and for pre- and post-education comparative testing.

## Conclusion

We have presented the results from the first phase in the development and testing of the GPACS. Phase one results suggest that a 20-item measure (four items removed based on exclusion criteria) is reliable and valid when administered to a sample of medically trained individuals. In its current format, the GPACS-D-D displays acceptable reliability and validity and is suitable for administration as a measure of attitude and confidence change before and after targeted dementia education with analysis permissible at the item level. Theorised relationships between attitude, confidence, and behaviour suggest that improving GP knowledge through targeted education may affect clinical behaviour mediated by attitude and confidence. In this way, changes in confidence and attitude may indicate intention to change one’s behaviour and signal potential improvements in clinical care for people with dementia.

## Abbreviations

CFA:, confirmatory factor analysis; GP:, general practitioner; GPACS-D:, General Practitioner Attitudes and Confidence Scale-Dementia; PCA:, principal component analysis.

## References

[1] Perry M, Draskovic I, van Achterberg T, Borm GF, van Eijken MIJ, Lucassen PL, Vernooij-Dassen MJFJ, Olde Rikkert MGM. Can an EASY care based dementia training programme improve diagnostic assessment and management of dementia by general practitioners and primary care nurses? the design of a randomised controlled trial. BMC Health Serv Res. 2008;8:71. doi:10.1186/1472-6963-8-71. 10.1186/1472-6963-8-71 PMC2391160 18384675

[2] Milne AJ, Hamilton-West K, Hatzidimitriadou E (2005). GP attitudes to early diagnosis of dementia: evidence of improvement. Aging Ment. Health.

[3] Meuser TM, Boise L, Morris JC (2004). Clinical benefits and practices in dementia care: implications for health educators. Educ. Gerontol..

[4] Hansen EC, Hughes C, Routley G, Robinson AL (2008). General practitioners’ experiences and understandings of diagnosing dementia: factors impacting on early diagnosis. Soc. Sci. Med..

[5] Bamford C, Lamont S, Eccles M, Robinson L, May C, Bond J (2004). Disclosing a diagnosis of dementia: a systematic review. Int J Geriatr Psychiatry..

[6] Carpenter B, Dave J (2004). Disclosing a dementia diagnosis: a review of opinion and practice, and a proposed research agenda. Gerontologist.

[7] Boise L, Camicioli R, Morgan DL, Rose JH, Congleton L (1999). Diagnosing dementia: perspectives of primary care physicians. Gerontologist.

[8] Fabrigar LR, Smith SM, Petty RE, Crites SLJ (2006). Understanding knowledge effects on attitude-behavior consistency: the role of relevance, complexity, and amount of knowledge. J. Pers. Soc. Psychol..

[9] Ajzen I (1991). The theory of planned behavior. Organ. Behav. Hum. Decis. Process..

[10] Bandura A (2001). Social cognitive theory: an agentic perspective. Annu. Rev. Psychol..

[11] Hendrick TAM, Fischer ARH, Tobi H, Frewer LJ (2013). Self reported attitude scales: current practice in adequate assessment of reliability, validity, and dimensionality. J. Appl. Soc. Psychol..

[12] Ajzen I, Madden TJ (1985). Prediction of goal-directed behavior: attitudes, intentions, and perceived behavioral control. J. Exp. Soc. Psychol..

[13] Phillips J, Pond D, Goode S (2011). Timely diagnosis of dementia: can we do better.

[14] Cahill S, Clark M, O’Connell H, Lawlor B, Coen RF, Walsh C (2008). The attitudes and practices of general practitioners regarding dementia diagnosis in Ireland. Int J Geriatr Psychiatry..

[15] Kaduszkiewicz H, Wiese B, van den Bussche H (2008). Self-reported competence, attitude and approach of physicians towards patients with dementia in ambulatory care: results of a postal survey. BMC Health Serv. Res..

[16] Turner S, Iliffe S, Downs M, Wilcock J, Bryans M, Levin E, Keady J, O’Carroll R (2004). General practitioners’ knowledge, confidence and attitudes in the diagnosis and management of dementia. Age Ageing.

[17] Annear M, Toye C, Eccleston C, McInerney F, Elliott K, Tranter B, Hartley T, Robinson A (2015). Dementia Knowledge Assessment Scale (DKAS): development and preliminary psychometric properties. J. Am. Geriatr. Soc..

[18] Leung JLM, Sezto NW, Chan WC, Cheng SP, Tabg SH, Lam LCW (2013). Attitudes and perceived competence of residential care homes staff about dementia care. Asian J Gerontol Geriatr..

[19] Cahill S, Clark M, Walsh C, O’Connell H, Lawlor B (2006). Dementia in primary care: the first survey of Irish general practitioners. Int J Geriatr Psychiatry..

[20] O’Connor ML, McFadden SH (2010). Development and psychometric validation of dementia attitudes scale. Int. J. Alzheimers Dis..

[21] Corp IBM (2011). IBM SPSS Statistics for Windows, Version 20.0.

[22] Svensson B, Markstrom U, Bejerholm U, Bjorkman T, Brunt D, Eklund M, Hansson L, Leufstadius C, Gyllensten AL, Sandlund M (2011). Test - retest reliability of two instruments for measuring public attitudes towards persons with mental illness. BMC Psychiatry.

[23] Sim J, Wright CC (2005). The kappa statistic in reliability studies: use, interpretation, and sample size requirements. Phys. Ther..

[24] Kottner J, Audige L, Brorson S, Donner A, Gajewski BJ, Hrobjartsson A, Roberts C, Shoukri M, Streiner DL (2011). Guidelines for Reporting Reliability and Agreement Studies (GRRAS) were proposed. J. Clin. Epidemiol..

[25] Fleiss JL, Cohen J (1973). The equivalence of weighted kappa and the intraclass correlation coefficient as measures of reliability. Educ. Psychol. Meas..

[26] Cicchetti DV (2010). Methodological commentary the precision of reliability and validity estimates revisited: distinguishing between clinical and statistical significance of sample size requirements. J Clin Exp Neuropshchol.

[27] Field A (2013). Discovering statistics using IBM SPSS statistics.

[28] Liu JY, Lai C, Dai D, Ting S, Choi K (2013). Attitudes in the management of patients with dementia: comparison in doctors with and without special training. East Asian Arch Psychiatry..

